# Supplementary material for: Electrolytes as predictors of fibro fatigue scores in Long-COVID patients
Source: PLoS One. 2024 Aug 28;19(8):e0309348. doi: 10.1371/journal.pone.0309348 (PMC11357109; doi:10.1371/journal.pone.0309348)
Supplement: Attachment — Submitted filename: Response to reviewers.docx [file pone.0309348.s001.docx]

Response to reviewers

PONE-D-24-09563

Electrolytes as predictors of Fibro Fatigue (FF) scores in Long COVID patients: A focus on Calcium and Magnesium

PLOS ONE

Dear Editor,

 Thank you very much for your effort and to the reviewers for reviewing our manuscript and adding important notes and requirements. We responded positively to all the editor's and reviewers' concerns. We highlighted the amendments in the revised manuscript along with our response to reviewers below. Our responses are in RED.

Thank you again and accept my best regards.

Yours

Wasim T. Al Masoodi

The Corresponding Author

==============================
**ACADEMIC EDITOR:**

1. You have to be consistent to say COVID-19 because I saw some COVID-19 and some COVID.

RESPONSE: The disease caused by the infection with the SARS-COV-2 is called COVID-19. In comparison, the persistent symptoms syndrome is called long-COVID without number 19. However, we checked the whole manuscript and corrected any missing words. Thank you.

2. You need to revise the margin type of references
3. RESPONSE: We revised the margin type of references. They were made automatically by ENDNOTE software. Thank you.
4. You need to explain 1 or 2 sentences about the table and figure.
5. RESPONSE: Thank you for your suggestion. We added statements explaining the tables and figures with references supporting our explanation.

==============================

**Journal Requirements:**

When submitting your revision, we need you to address these additional requirements.

1. Please ensure that your manuscript meets PLOS ONE's style requirements, including those for file naming. The PLOS ONE style templates can be found at
   <https://journals.plos.org/plosone/s/file?id=wjVg/PLOSOne_formatting_sample_main_body.pdf> and 
   <https://journals.plos.org/plosone/s/file?id=ba62/PLOSOne_formatting_sample_title_authors_affiliations.pdf>

RESPONSE: Yes, we followed the requirements exactly. Thank you.

2. We note that your Data Availability Statement is currently as follows: All relevant data are within the manuscript and its Supporting Information files.

Please confirm at this time whether or not your submission contains all raw data required to replicate the results of your study. Authors must share the “minimal data set” for their submission. PLOS defines the minimal data set to consist of the data required to replicate all study findings reported in the article, as well as related metadata and methods (<https://journals.plos.org/plosone/s/data-availability#loc-minimal-data-set-definition>).

For example, authors should submit the following data:

- The values behind the means, standard deviations and other measures reported;
- The values used to build graphs;
- The points extracted from images for analysis.

Authors do not need to submit their entire data set if only a portion of the data was used in the reported study.

If your submission does not contain these data, please either upload them as Supporting Information files or deposit them to a stable, public repository and provide us with the relevant URLs, DOIs, or accession numbers. For a list of recommended repositories, please see <https://journals.plos.org/plosone/s/recommended-repositories>.

If there are ethical or legal restrictions on sharing a de-identified data set, please explain them in detail (e.g., data contain potentially sensitive information, data are owned by a third-party organization, etc.) and who has imposed them (e.g., an ethics committee). Please also provide contact information for a data access committee, ethics committee, or other institutional body to which data requests may be sent. If data are owned by a third party, please indicate how others may request data access.

RESPONSE: We declare that all raw data required to replicate the results of our study. Thank you.

2. When completing the data availability statement of the submission form, you indicated that you will make your data available on acceptance. We strongly recommend all authors decide on a data sharing plan before acceptance, as the process can be lengthy and hold up publication timelines. Please note that, though access restrictions are acceptable now, your entire data will need to be made freely accessible if your manuscript is accepted for publication. This policy applies to all data except where public deposition would breach compliance with the protocol approved by your research ethics board. If you are unable to adhere to our open data policy, please kindly revise your statement to explain your reasoning and we will seek the editor's input on an exemption. Please be assured that, once you have provided your new statement, the assessment of your exemption will not hold up the peer review process.

RESPONSE: Our data will be available after acceptance upon a suitable request from the journal. Thank you.

3. Please amend either the title on the online submission form (via Edit Submission) or the title in the manuscript so that they are identical.

RESPONSE: Sorry for this error. We use one title for the submission. Thank you.

6. Please ensure that you include a title page within your main document. You should list all authors and all affiliations as per our author instructions and clearly indicate the corresponding author.

RESPONSE: Yes, we added the title page in the main document with all required information. Thank you.

7. Please include your tables as part of your main manuscript and remove the individual files. Please note that supplementary tables (should remain/ be uploaded) as separate "" supporting information"" files

RESPONSE: Yes, we included tables as part of the whole manuscript and removed the individual files. Thank you.

8. Please include captions for your Supporting Information files at the end of your manuscript, and update any in-text citations to match accordingly. Please see our Supporting Information guidelines for more information: <http://journals.plos.org/plosone/s/supporting-information>.

RESPONSE: We have no supplementary files. Thank you.

8. Please review your reference list to ensure that it is complete and correct. If you have cited papers that have been retracted, please include the rationale for doing so in the manuscript text, or remove these references and replace them with relevant current references. Any changes to the reference list should be mentioned in the rebuttal letter that accompanies your revised manuscript. If you need to cite a retracted article, indicate the article’s retracted status in the References list and also include a citation and full reference for the retraction notice.

RESPONSE: We reviewed the reference list and corrected it as required. No retracted references are included. Thank you.

**Additional Editor Comments:**

Electrolytes as predictors of Fibro Fatigue (FF) scores in Long COVID patients: A focus on Calcium and Magnesium

1. You have to be consistence to say COVID-19 because I saw some COVID-19 and some COVID
2. You need to revise the margin type of references
3. You need to explain 1 or 2 sentences about the table and figure.


[Note: HTML markup is below. Please do not edit.]

Reviewers' comments:

Reviewer's Responses to Questions

RESPONSE: We corrected all the above concerns as seen above. Thank you.

**Comments to the Author**

1. Is the manuscript technically sound, and do the data support the conclusions?

The manuscript must describe a technically sound piece of scientific research with data that supports the conclusions. Experiments must have been conducted rigorously, with appropriate controls, replication, and sample sizes. The conclusions must be drawn appropriately based on the data presented.

Reviewer #1: Yes

Reviewer #3: Yes

2. Has the statistical analysis been performed appropriately and rigorously?

Reviewer #1: Yes

Reviewer #3: Yes

3. Have the authors made all data underlying the findings in their manuscript fully available?

The [PLOS Data policy](http://www.plosone.org/static/policies.action#sharing) requires authors to make all data underlying the findings described in their manuscript fully available without restriction, with rare exception (please refer to the Data Availability Statement in the manuscript PDF file). The data should be provided as part of the manuscript or its supporting information, or deposited to a public repository. For example, in addition to summary statistics, the data points behind means, medians and variance measures should be available. If there are restrictions on publicly sharing data—e.g. participant privacy or use of data from a third party—those must be specified.

Reviewer #1: Yes

Reviewer #3: Yes

4. Is the manuscript presented in an intelligible fashion and written in standard English?

PLOS ONE does not copyedit accepted manuscripts, so the language in submitted articles must be clear, correct, and unambiguous. Any typographical or grammatical errors should be corrected at revision, so please note any specific errors here.

Reviewer #1: Yes

Reviewer #3: Yes

5. Review Comments to the Author

Please use the space provided to explain your answers to the questions above. You may also include additional comments for the author, including concerns about dual publication, research ethics, or publication ethics. (Please upload your review as an attachment if it exceeds 20,000 characters)

Reviewer #1: The presented topic is significant, and the authors used electrolyte levels to predict long-term COVID-19 patients with high FF levels and discussed the observed differences in biochemical and clinical profiles highlight the widespread impact of FF on Long-COVID people's health outcomes. They have proven their claims through the results provided with statistical operations and performed the ROC-AUC analysis However, I have one concern that the authors have to consider and resolve, as follows:
1. At the end of the introduction part, there is no general overview of the arrangement of the rest of the manuscript; it should provide an overview of the following sections of the manuscript.

RESPONSE: We added a general overview of the arrangement of the rest of the manuscript. Thank you.

Reviewer #3: Q- Were there any notable biases or limitations in the selection of participants, such as age distribution or comorbidities?

RESPONSE: We added a statement explaining the selection of participants in the “Subjects” section. Thank you.

Q- Were there any control measures taken to minimize confounding factors that could influence the results?

RESPONSE: Yes, we deliberately choose the control group and excluded many of them in order to obtain a group that has no significant difference in the cofounders such as marital status, BMI, age, smoking, comorbidities. This selection would produce a conclusion that any difference will be due to the disease only; not due to other cofounders. Thank you.

Q- What are the potential clinical implications of the study findings for managing Long-COVID patients with fibro-fatigue?

RESPONSE: We added a statement explaining the importance of the results of the study in management of Patients with FF. Thank you.

Q- How might the predictive biomarkers identified in this study impact clinical practice or patient care strategies?

RESPONSE: We included a statement elucidating the significance of the study's findings in the clinical state care of the Long-COVID patients with severe FF. Thank you.

Q- Are there any recommendations for further research or validation studies based on the findings?

RESPONSE: Yes, the recommendation for the future work has been added at the end of discussion section. Thank you.
